# *FTO* and *MC4R* Gene Variants Are Associated with Obesity in Polycystic Ovary Syndrome

**DOI:** 10.1371/journal.pone.0016390

**Published:** 2011-01-20

**Authors:** Kathryn G. Ewens, Michelle R. Jones, Wendy Ankener, Douglas R. Stewart, Margrit Urbanek, Andrea Dunaif, Richard S. Legro, Angela Chua, Ricardo Azziz, Richard S. Spielman, Mark O. Goodarzi, Jerome F. Strauss

**Affiliations:** 1 Department of Genetics, University of Pennsylvania School of Medicine, Philadelphia, Pennsylvania, United States of America; 2 Division of Cancer Epidemiology and Genetics, Clinical Genetics Branch, National Cancer Institute, Rockville, Maryland, United States of America; 3 Division of Endocrinology, Metabolism and Molecular Medicine, Northwestern University Feinberg School of Medicine, Chicago, Illinois, United States of America; 4 Department of Obstetrics and Gynecology, Penn State Hershey College of Medicine, Hershey, Pennsylvania, United States of America; 5 Division of Endocrinology, Diabetes, and Metabolism, Cedars-Sinai Medical Center, Los Angeles, California, United States of America; 6 Department of Obstetrics and Gynecology, Cedars-Sinai Medical Center, Los Angeles, California, United States of America; 7 Department of Obstetrics and Gynecology, Virginia Commonwealth University, Richmond, Virginia, United States of America; Cardiff University, United Kingdom

## Abstract

Polycystic ovary syndrome (PCOS) is the leading cause of anovulatory infertility in women. It is also associated with metabolic disturbances that place women at increased risk for obesity and type 2 diabetes. There is strong evidence for familial clustering of PCOS and a genetic predisposition. However, the gene(s) responsible for the PCOS phenotypes have not been elucidated. This two-phase family-based and case-control genetic study was designed to address the question of whether SNPs identified as susceptibility loci for obesity in genome-wide association studies (GWAS) are also associated with PCOS and elevated BMI. Members of 439 families having at least one offspring with PCOS were genotyped for 15 SNPs previously shown to be associated with obesity. Linkage and association with PCOS was assessed using the transmission/disequilibrium test (TDT). These SNPs were also analyzed in an independent case-control study involving 395 women with PCOS and 176 healthy women with regular menstrual cycles. Only one of these 15 SNPs (rs2815752 in *NEGR1*) was found to have a nominally significant association with PCOS (χ^2^ = 6.11, P = 0.013), but this association failed to replicate in the case-control study. While not associated with PCOS itself, five SNPs in FTO and two in MC4R were associated with BMI as assessed with a quantitative-TDT analysis, several of which replicated association with BMI in the case-control cohort. These findings demonstrate that certain SNPs associated with obesity contribute to elevated BMI in PCOS, but do not appear to play a major role in PCOS *per se*. These findings support the notion that PCOS phenotypes are a consequence of an oligogenic/polygenic mechanism.

## Introduction

Polycystic ovary syndrome (PCOS) is a common endocrine disorder affecting 5–8% of reproductive age women. In addition to the primary features of hyperandrogenemia, chronic anovulation and infertility, women with PCOS are also at increased risk for obesity, insulin resistance and type 2 diabetes. Forty to 80% of women with PCOS are overweight or obese [1]–[3]. Moreover, in obese women the reproductive phenotype of PCOS can be reversed by weight loss [4], implicating BMI as a major determinant of the manifestation of the syndrome. Collectively, these observations strongly suggest that obesity and PCOS are linked co-morbidities. Furthermore, because of the common co-occurrence of these features, PCOS casts a shadow well beyond the reproductive years when it is diagnosed.

Legro et al. [5] described evidence for familial aggregation of hyperandrogenemia in PCOS that is consistent with a genetic contribution to disease susceptibility. However, the diagnostic criteria used for PCOS in the Legro et al. study (hyperandrogenemia and oligomenorrhea/amenorrhea), and others that have presented strong evidence for familial clustering do not include metabolic phenotypes such as obesity or indices of glucose metabolism or insulin action. Few genetic studies on PCOS have focused on obesity. Consequently the contribution of genes that influence body composition in PCOS remains to be clarified.

Recent technological and computational advances in genome-wide association studies (GWAS) have resulted in a series of studies designed to identify susceptibility loci for many complex genetic diseases, including obesity and type 2 diabetes. These large scale genetic association studies have identified variations in or near *FTO, GNPDA2, INSIG2, KCTD15, MC4R, MTCH2, NEGR1, SH2B1* and *TMEM18* as susceptibility loci for obesity [6]–[10].

Given the high prevalence of obesity in women with PCOS, it is crucial to investigate the genetic contribution the susceptibility loci for obesity make to PCOS. A study by Barber et al. [11] identified an association between the *FTO* SNP rs9939609 and PCOS, but determined that its effect was likely through BMI. Similarly, Tan et al. [12] and Wehr et al. [3] also demonstrated an association between BMI and rs9939609 in women with PCOS. In this report we examined SNPs in or near a set of nine genes associated with obesity based on findings from GWAS or large association studies.

## Materials and Methods

### PCOS families

SNP markers were genotyped in 439 families with PCOS: 44 multiplex families (two or more affected daughters and parents) and 395 simplex families (one affected daughter and parents). The total number of offspring with PCOS was 488. Body mass index (BMI) was available for 463 of the PCOS probands and sisters. Clinical characteristics of the probands and sisters are presented in Table 1. The self-identified ethnicities of probands in the families were: 87% white, 4% Hispanic, 1% black and 7% other or unknown.

**Table 1:** Clinical characteristics of PCOS in probands and sisters in the family study, controls from the case-control cohort, and PCOS cases from the case-control cohort.

	PCOS probands and sisters (N = 488)	Controls (N = 176)	PCOS cases (N = 395)
Age (yr)	28 (24–32)	34 (26–41)	27 (22–32) b
BMI (kg/m^2^)	35.3 (28.7–41.1)	24.1 (21.9–28.5)	31.5 (25.1–39.7) a b
Total testosterone (nmol/l)*	2.46 (2.04–3.19)	1.39 (0.97–1.91)	2.39 (1.66–3.12) b c
Insulin (pmol/l)	152.8 (97.2–215.3)	50.9 (33.9–81.4)	100.5 (43.1–165.0) a b
Glucose (mmol/l)	4.88 (4.61–5.27)	4.72 (4.52–5.08)	4.72 (4.44–5.05) a
HOMA2-IR	2.72 (1.82–3.84)	0.98 (0.66–1.53)	1.72 (0.81–2.76) a b
HOMA2-%B	195.9 (152.8–240.9)	105.4 (79.4–135.5)	147.8 (99.8–200.6) a b

Data are median (interquartile range).

*Nominal testosterone values based on different assays for the probands and sisters and the case-control cohort as described in text.

a P<0.0001 compared to probands and sisters.

b P<0.0001 compared to controls.

c P = 0.002 compared to probands and sisters.

Diagnostic criteria for PCOS have been described in detail elsewhere [5], [13]. Probands and sisters were considered affected if they had 6 or fewer menses per year and elevated total testosterone (greater than 58 ng/dl) or elevated non-SHBG-bound testosterone (greater than 15 ng/dl); these thresholds are 2 S.D. greater than the mean of our normal controls.

### Case-control cohort

The replication cohort consisted of 395 unrelated White PCOS patients and 176 White control women recruited at two centers, the University of Alabama at Birmingham (248 PCOS and 152 controls) and Cedars-Sinai Medical Center (147 PCOS and 24 controls). Cases were premenopausal, non-pregnant, on no hormonal therapy, including oral contraceptives, for at least three months, and met 1990 NIH criteria for PCOS [14]. Parameters for defining hirsutism, hyperandrogenemia, ovulatory dysfunction, and exclusion of related disorders were previously reported [15]. Clinical characteristics of the case-control cohort are presented in Table 1. Controls were healthy women, with regular menstrual cycles and no evidence of hirsutism, acne, alopecia, or endocrine dysfunction and had not taken hormonal therapy (including oral contraceptives) for at least three months.

This study was approved by the institutional review boards of the University of Pennsylvania, Pennsylvania State University College of Medicine, Brigham and Women's Hospital, Northwestern University, the University of Alabama at Birmingham and Cedars-Sinai Medical Center. All subjects provided written informed consent for participation in this study.

### SNP genotyping

SNPs were chosen for genotyping in the PCOS families on the basis of published findings in GWA studies with obesity. A total of 15 SNPs associated with obesity that were genotyped are located in or near *FTO* (rs1421085, rs17817449, rs8050136, rs9939609 and rs9930506) [9], [10], [16], *GNPDA2* (rs10938397), *KCTD15* (rs11084753), *MTCH2* (rs10838738), *NEGR1* (rs2815752), *SH2B1* (rs7498665), *TMEM1*8 (rs6548238) [9], *MC4R* (rs17782313 and rs12970134) [7], [9], [17], and in *INSIG2* (rs7566605 and rs2161829) [6]. SNPs were genotyped using Applied Biosystems TaqMan SNP Genotyping Assays. Allelic PCR products were analyzed using the Applied Biosystems 7900HT Sequence Detection System and SDS 2.2 software. Genotypes were auto-called by SDS 2.2 software with quality value set at 0.95. Two CEPH individuals were typed on each of 16 96-well plates. No discrepancies were observed for any of the SNPs, and all genotypes were in Hardy-Weinberg equilibrium.

In the case-control cohort, genotyping was carried out using iSelect Infinium technology, following the manufacturer's protocol (Illumina, San Diego, CA) [18], [19]. Duplicate genotyping of 12 samples yielded a 100% concordance rate. The genotyping success rate was 99.97%. All SNPs were in Hardy-Weinberg equilibrium. SNPs were excluded if the genotyping failure rate was >10%; or if the minor allele frequency was <3%. Ultimately, of the 15 SNPs genotyped in the family cohort, 13 were genotyped in the case-control cohort.

### Statistical analysis

Error-checking of genotypes in the family material was performed with Merlin software (version 1.1.2; http://www.sph.umich.edu/csg/abecasis/merlin/index.html [20]) and families with one or more Mendelian discrepancies for a marker were excluded in the analysis of that markerLinkage and association between SNPs and PCOS was tested with the TDT [21]. Odds ratio (OR) and standard error (SE) was calculated using the method described in Kazeem and Farrall [22]. The quantitative TDT (QTDT) program (version 2.6.0) in Merlin was used to test for association between the SNPs and BMI, using the orthogonal association model and the environmental, polygenic, and additive variance components (http://www.sph.umich.edu/csg/abecasis/QTDT/) [20]). We corrected for multiple testing using Bonferroni adjustment based on testing of 15 SNPs associated with obesity; the adjusted p-value of 0.0033 corresponded to a nominal P = 0.05.

In the case-control cohort, genotypic association with PCOS status was evaluated using logistic regression, adjusting for recruitment site, BMI and age. In women with PCOS, association between genotype and BMI was performed using linear regression adjusting for site and age. Additive, dominant, and recessive models were examined. A P<0.05 was considered significant when there was evidence of association in the family cohort. For other SNPs, Bonferroni-corrected P value of 0.0038, corresponding to a nominal P of 0.05 was utilized.

Genetic Power Calculator software (http://pngu.mgh.harvard.edu/~purcell/gpc/ [23]) was used to determine that with the sample size of each independent cohort, there was approximately 80% power (P = 0.05) to detect a relative risk ration of 3.0.

## Results

To determine if sequence variants in genes associated with obesity contribute to genetic susceptibility to PCOS, we used the TDT to analyze 15 SNPs in nine genes for association with PCOS in our collection of 439 families (Table 2). The TDT analysis was nominally significant for only one SNP: rs2815752 in *NEGR1* (χ^2^ = 6.11, P = 0.013), which was not significant after adjustment for multiple testing, nor was it significant in an independent case-control analysis (Table 2).

**Table 2:** Obesity susceptibility loci identified in GWAS tested by TDT and QTDT 439 PCOS families (N = 488 probands and sisters) for linkage and association with PCOS and BMI.

				TDT association with PCOS									QTDT association with BMI	
Gene	SNP	Alleles^a^	MAF^b^	Over-transmittedAllele	T^c^	not T^c^	Total T^c^	TransmissionFrequency	TDTχ ^2^	P-value^d^	OR	SE	QTDTχ ^2^	P-value^e^
*FTO*	rs1421085	T/C	0.45	C	232	195	427	0.543	3.21	0.073	1.178	0.096	11.42	**0.0007**
	rs17817449	T/G	0.453	G	235	214	449	0.523	0.98	0.322	1.081	0.093	10.25	**0.0014**
	rs8050136	C/A	0.451	A	247	227	474	0.521	0.84	0.358	1.081	0.091	7.22	**0.0072**
	rs9939609	T/A	0.456	A	235	223	458	0.513	0.31	0.575	1.043	0.092	5.30	**0.0213**
	rs9930506	A/G	0.467	G	229	213	442	0.518	0.58	0.447	1.068	0.094	8.98	**0.0027**
*GNPDA2*	rs10938397	A/G	0.427	G	201	200	401	0.501	0.002	0.964	1.025	0.099	0.67	0.4131
*INSIG2*	rs7566605	G/C	0.314	G	108	104	212	0.509	0.06	0.784	1.048	0.136	0.23	0.6315
	rs2161829	G/A	0.417	A	126	120	246	0.512	0.15	0.702	1.041	0.127	0.42	0.5169
*KCTD15*	rs11084753	G/A	0.336	G	204	196	400	0.510	0.16	0.689	1.055	0.099	1.37	0.2418
*MC4R*	rs17782313	T/C	0.257	C	167	142	309	0.540	2.02	0.155	1.207	0.112	6.56	**0.0104**
	rs12970134	G/A	0.28	A	164	148	312	0.526	0.82	0.365	1.132	0.111	7.81	**0.0052**
*MTCH2*	rs10838738	A/G	0.361	G	209	195	404	0.517	0.49	0.486	1.076	0.099	0.38	0.5376
*NEGR1*	rs2815752	A/G	0.352	A	221	172	393	0.562	6.11	**0.013**	1.285	0.100	0.81	0.3681
*SH2B1*	rs7498665	A/G	0.381	G	194	189	383	0.507	0.07	0.799	1.010	0.101	1.76	0.1846
*TMEM18*	rs6548238	C/T	0.172	C	139	130	269	0.517	0.30	0.583	1.092	0.121	0.23	0.6315

a SNP alleles, minor allele appears second.

b MAF, minor allele frequency for SNP.

c T, number of transmissions to affected offspring in the TDT analysis.

d P values are uncorrected.

Since all 15 SNPs have been associated with obesity in the general population, we carried out TDT analysis separately in obese (BMI>30 kg/m^2^) and non-obese (BMI<29.9 kg/m^2^) offspring to determine whether any of them were associated with PCOS in each BMI stratum. ne SNP in *FTO*, two in *MC4R* and one in *NEGR1* were over-transmitted to the obese offspring (*FTO* rs1421085: χ^2^ = 4.99, P = 0.026; *MC4R* rs17782313 and rs12970134: χ^2^ = 8.24, P = 0.004 and χ^2^ = 5.065, P = 0.024, respectively; *NEGR1* rs2815752: χ^2^ = 6.26, P = 0.012). However, none were significant after correction for multiple testing.

QTDT analysis further revealed that five SNPs in *FTO* and two in *MC4R* were significantly associated with obesity in the PCOS families (Table 2). Of these, three SNPs in *FTO* remained significant after correction for multiple testing: rs1421085 (P_corrected_  = 0.011), rs17817449 (0.021), and rs9930506 (0.041).

We next investigated association between 13 of these 15 SNPs and BMI in an independent case-control study. None of these SNPs was associated with PCOS (Table 3). No SNPs were associated with PCOS in analyses stratified by obesity.

**Table 3:** Obesity susceptibility loci identified in GWAS tested by logistic regression for association with PCOS in the case-control cohort (395 cases, 176 controls).

			ADDITIVE				DOMINANT				RECESSIVE			
Gene	SNP	Minor allele	N	OR	STAT	P	N	OR	STAT	P	N	OR	STAT	P
*FTO*	rs1421085	C	571	0.830	−1.080	0.280	571	0.633	−1.819	0.069	571	1.134	0.385	0.700
	rs17817449	G	571	0.834	−1.040	0.298	571	0.646	−1.725	0.084	571	1.116	0.337	0.736
	rs8050136	A	571	0.842	−0.985	0.325	571	0.657	−1.670	0.095	571	1.134	0.382	0.702
	rs9939609	A	571	0.820	−1.137	0.256	571	0.635	−1.799	0.072	571	1.088	0.258	0.796
	rs9930506	G	571	0.844	−0.972	0.331	571	0.642	−1.686	0.092	571	1.103	0.318	0.751
*GNPDA2*	rs10938397	G	571	1.141	0.775	0.439	571	1.020	0.082	0.935	571	1.536	1.316	0.188
*KCTD15*	rs11084753	A	570	1.345	1.662	0.096	570	1.607	2.053	0.040	570	1.088	0.220	0.826
*MC4R*	rs17782313	C	571	1.341	1.459	0.145	571	1.450	1.564	0.118	571	1.259	0.410	0.682
	rs12970134	A	571	1.377	1.645	0.100	571	1.619	2.041	0.041	571	0.979	−0.044	0.965
*MTCH2*	rs10838738	G	571	1.062	0.359	0.720	571	1.044	0.189	0.851	571	1.171	0.454	0.650
*NEGR1*	rs2815752	G	571	0.953	−0.294	0.769	571	0.962	−0.167	0.867	571	0.898	−0.342	0.732
*SH2B1*	rs7498665	G	571	0.947	−0.326	0.744	571	1.011	0.046	0.963	571	0.788	−0.717	0.474
*TMEM18*	rs6548238	T	571	0.817	−0.949	0.343	571	0.728	−1.304	0.192	571	1.599	0.634	0.526

In additive and recessive models, four of the five *FTO* SNPs were associated with BMI in women with PCOS (Table 4). One of the two *MC4R* SNPs was associated with BMI in the recessive model (Table 4). None of these *FTO* or *MC4R* SNPs was associated with BMI in the control group.

**Table 4:** Obesity susceptibility loci identified in GWAS tested by linear regression for association with BMI in 395 women with PCOS.

			ADDITIVE				DOMINANT				RECESSIVE			
Gene	SNP	Minor allele	N	BETA	STAT	P	N	BETA	STAT	P	N	BETA	STAT	P
*FTO*	rs1421085	C	395	0.049	2.521	**0.012**	395	0.040	1.391	0.165	395	0.101	2.876	**0.004**
	rs17817449	G	395	0.043	2.188	**0.029**	395	0.033	1.148	0.252	395	0.092	2.569	**0.011**
	rs8050136	A	395	0.046	2.362	**0.019**	395	0.038	1.312	0.190	395	0.095	2.684	**0.008**
	rs9939609	A	395	0.047	2.412	**0.016**	395	0.040	1.385	0.167	395	0.095	2.684	**0.008**
	rs9930506	G	395	0.024	1.227	0.221	395	0.014	0.485	0.628	395	0.056	1.606	0.109
*GNPDA2*	rs10938397	G	395	0.034	1.726	**0.085**	395	0.056	1.894	0.059	395	0.028	0.843	0.400
*KCTD15*	rs11084753	A	394	−0.014	−0.666	0.506	394	−0.030	−1.112	0.267	394	0.013	0.319	0.750
*MC4R*	rs17782313	C	395	0.026	1.155	0.249	395	0.024	0.887	0.376	395	0.064	1.108	0.268
	rs12970134	A	395	0.041	1.940	0.053	395	0.031	1.152	0.250	395	0.118	2.428	**0.016**
*MTCH2*	rs10838738	G	395	0.005	0.239	0.811	395	0.021	0.762	0.447	395	−0.025	−0.625	0.532
*NEGR1*	rs2815752	G	395	−0.024	−1.187	0.236	395	−0.006	−0.225	0.823	395	−0.082	−2.069	**0.039**
*SH2B1*	rs7498665	G	395	0.007	0.343	0.732	395	0.016	0.568	0.570	395	−0.005	−0.124	0.901
*TMEM18*	rs6548238	T	395	0.019	0.773	0.440	395	0.009	0.309	0.758	395	0.117	1.542	0.124

It should be noted that the affected women in the family cohort were heavier, more hyperandrogenemic based on nominal testosterone values, and had more prominent metabolic abnormalities than the PCOS women from the case-control cohort, which may be due to the fact that the former were all hyperandrogenemic by definition and, therefore, had a more profound PCOS phenotype (Table 1).

## Discussion

In this study, we examined whether DNA variants that contribute to obesity also influence susceptibility to PCOS. This study was designed to address the question of whether the frequent co-occurrence of obesity with PCOS might be due to underlying genetic mechanisms that are common to these conditions or whether they are separate and independent. The initial phase of this study was a family based analysis followed by confirmation of the findings in an independent case-control cohort. Ideally, women with PCOS in both cohorts would have been diagnosed using the same criteria. This was not the case because these are both pre-existing cohorts. Despite this difference several *FTO* SNPs were associated with BMI in both studies.

Among the 15 obesity-associated SNPs that were assessed by family-based TDT analysis for association with PCOS, rs2815752 near *NEGR1* was nominally significant (P = 0.013). Several SNPs in *FTO* which are not significantly associated with PCOS, were associated with obesity in affected women in both the family and case-control cohorts. Other studies have also found a similar association between DNA variation in *FTO* and PCOS. Barber et al [11] reported an association between the *FTO* SNP rs9939609 and PCOS, which became less significant after adjustment for BMI. In studies by Tan et al. [12] and Wehr et al. [3], association between SNPs in *FTO* and PCOS phenotype was not considered, but both reported an association of the rs9939609-allele A with increased BMI. These results suggest that variation in *FTO* is associated with obesity in PCOS, consistent with our findings, but that the contribution *FTO* makes to the PCOS reproductive phenotype is uncertain.

Our goal in this study was to determine whether specific SNPs associated with obesity in GWAS contributed to PCOS. Given the limited power in this study to detect SNPs with only a small effect (OR<3), we cannot rule out that these, or other SNPs in the same genes, may be found to be associated with PCOS in studies of larger cohorts. It is noteworthy that in a study of the contribution SNPs associated with type 2 diabetes make to PCOS, Biyasheva et al. [24] reported that while the two SNPs identified in GWAS were not significant in PCOS, two other SNPs mapping approximately 100 kb centrometic to them were significantly associated with PCOS.

Numerous candidate gene studies designed to identify PCOS susceptibility loci have been published, but most nominally significant positive findings have not been confirmed in follow-up replication studies (reviewed in [25], [26]). The most well established candidate region remains D19S884, a microsatellite marker in intron 55 of *FBN3*, located on 19p13.2 about 800 kb centrometic to the insulin receptor gene [13], [27]. However, there is no evidence to date that the D19S884 allele associated with PCOS influences insulin receptor gene expression, although it has been associated with insulin resistance [28]. Of relevance to the present study that revealed an association of MC4R SNPs with BMI, we recently found an association for SNPs in the gene that encodes the MC4R ligand, *POMC*, with PCOS [29]. Interestingly, humans with mutations in *POMC* and *MC4R* and mice with targeted deletions in these genes have an obesity phenotype [30], [31]. Thus, a neuroendocrine pathway may connect the reproductive and metabolic phenotypes found in women with PCOS.

Several but not all SNP associations observed in the family cohort were replicated in the case-control cohort. The relatively small number of subjects in the control group may have affected our ability to achieve replication. Thus, to more firmly rule out the non-replicated associations herein, replication efforts in much larger cohorts would be needed.

Given that PCOS is associated with obesity in a significant number of women, it is necessary to consider the interaction of genes underlying this complex phenotype. At one extreme would be non-overlapping sets of genes that predispose to PCOS and obesity. At the other extreme, one set of genes might contribute to both conditions (i.e. the underlying genetic predisposition is the same) with different environmental factors or modifiers triggering disease progression down one path or another. The third possibility is a combination of these two scenarios, with genes in pathways predisposing for obesity interacting or converging to enhance the risk of PCOS. The interaction between *MC4R* and *POMC* variants represents an example of this mechanism.

In conclusion, SNPs in *FTO* and *MC4R* were found to be associated with BMI in PCOS women, but appear not to contribute in a major way to the reproductive phenotypes of PCOS. However, these variants may interact with other genes (e.g., *POMC*) to predispose women to PCOS, consistent with the notion that PCOS is an oligogenic/polygenic disorder.
